# Effects of sex and APOE ε4 genotype on brain mitochondrial high-energy phosphates in midlife individuals at risk for Alzheimer’s disease: A ^31^Phosphorus MR spectroscopy study

**DOI:** 10.1371/journal.pone.0281302

**Published:** 2023-02-14

**Authors:** Steven Jett, Jonathan P. Dyke, Camila Boneu Yepez, Camila Zarate, Caroline Carlton, Eva Schelbaum, Grace Jang, Silky Pahlajani, Schantel Williams, Roberta Diaz Brinton, Lisa Mosconi

**Affiliations:** 1 Department of Neurology, Weill Cornell Medicine, New York, New York, United States of America; 2 Department of Radiology, Weill Cornell Medicine, New York, New York, United States of America; 3 Department of Pharmacology, University of Arizona, Tucson, Arizona, United States of America; 4 Department of Neurology, University of Arizona, Tucson, Arizona, United States of America; Preeminent Medical Phonics Education & Research Center, Hamamatsu University School of Medicine, JAPAN

## Abstract

Age, female sex, and APOE epsilon 4 (APOE4) genotype are the three greatest risk factors for late-onset Alzheimer’s disease (AD). The convergence of these risks creates a hypometabolic AD-risk profile unique to women, which may help explain their higher lifetime risk of AD. Less is known about APOE4 effects in men, although APOE4 positive men also experience an increased AD risk. This study uses ^31^Phosphorus Magnetic Resonance Spectroscopy (^31^P-MRS) to examine effects of sex and APOE4 status on brain high-energy phosphates [adenosine triphosphate (ATP), phosphocreatine (PCr), inorganic phosphate (Pi)] and membrane phospholipids [phosphomonoesters (PME), phosphodiesters (PDE)] in 209 cognitively normal individuals at risk for AD, ages 40–65, 80% female, 46% APOE4 carriers (APOE4+). Women exhibited lower PCr/ATP and PCr/Pi levels than men in AD-vulnerable regions, including frontal, posterior cingulate, lateral and medial temporal cortex (multi-variable adjusted p≤0.037). The APOE4+ group exhibited lower PCr/ATP and PCr/Pi in frontal regions as compared to non-carriers (APOE4-) (multi-variable adjusted p≤0.005). Sex by APOE4 status interactions were observed in frontal regions (multi-variable adjusted p≤0.046), where both female groups and APOE4+ men exhibited lower PCr/ATP and PCr/Pi than APOE4- men. Among men, APOE4 homozygotes exhibited lower frontal PCr/ATP than heterozygotes and non-carriers. There were no significant effects of sex or APOE4 status on Pi/ATP and PME/PDE measures. Among midlife individuals at risk for AD, women exhibit lower PCr/ATP (e.g. higher ATP utilization) and lower PCr/Pi (e.g. higher energy demand) than age-controlled men, independent of APOE4 status. However, a double dose of APOE4 allele shifted men’s brains to a similar metabolic range as women’s brains. Examination of brain metabolic heterogeneity can support identification of AD-specific pathways within at-risk subgroups, further advancing both preventive and precision medicine for AD.

## Introduction

Aging, female sex and Apolipoprotein E ε4 (APOE4) genotype are the strongest risk factors for late-onset Alzheimer’s disease (AD) [1]. The sex-based prevalence of AD is well documented with women accounting for over 60% of all those affected [2]. The lifetime risk of developing AD at age 45 is almost double in women than in men [2], an effect that is only partially explained by differences in survival rates and longevity [3, 4].

Additionally, sex differences in the effects of APOE4 on AD risk are well documented, with female carriers being affected earlier and in higher numbers than male carriers, though both sexes experience elevated risk relative to non-carriers [5, 6], a finding additionally dependent on age [7]. Further, APOE4 has a negative impact on AD biomarkers in a sex-dependent way, with generally larger risk estimates for women than men [8–13].

However, the interplay between age, chromosomal sex, and APOE4 genotype is complex, as multiple studies indicate differential effects of APOE4 allele dose on sex-related AD risk [5, 14–16]. Women heterozygous for APOE4 have a 4-fold increase in the risk of AD, whereas homozygous women and men with two copies of the APOE4 allele exhibit a 12-15-fold increase in risk as compared to non-carriers [5, 14–16].

The molecular mechanisms underlying the age, sex, and APOE4-linked heterogeneity in AD are under investigation. It has been proposed that this “triad of AD risk” exerts its actions via progressive alterations of coordinated systems biology events, especially bioenergetic cascades, that can span decades [1]. It is now accepted that the pathophysiological mechanisms of AD are activated years prior to clinically detectable symptoms and form the basis of a ~20 year prodromal phase of the disease [17]. Preclinical evidence indicates that all three major AD risk factors profoundly impact cerebral metabolic and bioenergetic processes starting in midlife, if not sooner, highlighting the role of mitochondrial function for AD risk [1, 18].

The brain is the most energetically demanding organ of the body, and is thus vulnerable to even modest declines in ATP generation [18]. Brain glucose hypometabolism, mitochondrial dysfunction, and reduced oxidative phosphorylation (OXPHOS) are consistent findings in AD [19–21]. A decline in mitochondrial function has been observed prior to the formation of amyloid-beta (Aβ) plaques, including decreased mitochondrial respiration, metabolic enzyme expression and activity, increased oxidative stress, and increased mitochondrial Aβ load [22–25].

Clinical ^18^F-fluoro-deoxygluose (FDG) Positron Emission Tomography (PET) studies also demonstrate reduced cerebral glucose metabolism (CMRglc) early in the course of AD [26, 27], especially among women at risk for AD [10, 28–32] and asymptomatic APOE4 carriers [33–36]. Consistent with preclinical work, in some studies, the extent of glucose hypometabolism exceeded that of Aβ load in at-risk women [10, 30]. However, the PET signal is based on trapping fluoro-deoxyglucose after phosphorylation to deoxy-glucose-6-phosphate, and therefore does not provide information on oxidative mitochondrial metabolism.

^31^Phosphorus Magnetic Resonance Spectroscopy (^31^P-MRS) is the only technique currently available that enables *in vivo* examination of brain mitochondrial function via mapping of intracellular high-energy phosphates (HEP), such as adenosine triphosphate (ATP), phosphocreatine (PCr), and inorganic phosphate (Pi) from mitochondria [37–39]. Using ^31^P-MRS, alterations in cerebral HEP levels have been reported in MCI and AD patients [40–42]. Alterations in ^31^P-MRS-derived phosphomonoesters (PME) and phosphodiesters (PDE) have also been noted [43, 44]. Currently, no studies have used ^31^P-MRS to test for metabolic abnormalities among asymptomatic, cognitively normal individuals at risk for AD.

Herein, we conducted a whole-brain, multi-slice ^31^P-MRS study to test for effects of sex and APOE4 status on HEP metabolites and membrane phospholipids in cognitively normal midlife men and women at risk for AD.

## Methods

### Participants and data

This is a natural history, non-interventional study of cognitively normal men and women ages 40–65 years, carrying risk factors for late-onset AD such as a family history and/or APOE4 genotype. Participants were recruited at the Weill Cornell Medicine (WCM) Alzheimer’s Prevention Program between 2018–2022 by self-referral, flyers, and word of mouth.

Our inclusion and exclusion criteria have been previously described [10, 28–32]. Briefly, all participants had Montreal Cognitive Assessment (MoCA) score≥26 and normal cognitive test performance by age and education [10, 28–32]. Exclusion criteria included medical conditions that may affect brain structure or function (e.g., stroke, any neurodegenerative diseases, major psychiatric disorders, hydrocephalus, demyelinating disorders such as Multiple Sclerosis, intracranial mass, and infarcts on MRI), use of psychoactive medications, and contraindications to MR imaging. All participants received medical, neurological, laboratory, cognitive and MRI exams, including volumetric MRI and ^31^P-MRS within 6 months of each other.

The patients’ sex was determined by self-report. A family history of late-onset AD was elicited using standardized questionnaires [10, 28–32]. APOE4 genotype was determined using standard qPCR procedures [10, 28–32]. Participants carrying one or two copies of the APOE4 allele were grouped together as APOE4 carriers (APOE4+), and compared to non-carriers (APOE4-).

### Standard protocol approvals, registrations, and patient consents

All methods were carried out in accordance with relevant guidelines and regulations. All experimental protocols were approved by the Weill Cornell Medicine Institutional Review Board. Written informed consent was obtained from all participants.

### Brain imaging

#### Image acquisition

All participants received a 3D volumetric T_1_-weighted MRI scan on a 3.0 T GE MR 750 Discovery scanner (General Electric, Waukesha, WI) [BRAVO; 1x1x1 mm resolution, 8.2 ms repetition time (TR), 3.2 ms echo time (TE), 12° flip angle, 25.6 cm field of view (FOV), 256x256 matrix with ARC acceleration] using a 32-channel head coil. The ^31^P-MRS scan was acquired on the same scanner as the MRI, typically on the same day, using a dual tuned ^31^P/^1^H quadrature head coil (Clinical MR Solutions, Brookfield, WI).

A 3 Plane Localizer image with 20 images in each orthogonal direction was acquired. Prior to MRS scanning, shimming was performed using a ^1^H single voxel technique placed over the entire brain avoiding the air-tissue interfaces. Shimming was done using ^1^H placement of a box in the cerebrum avoiding the hard palate and air/tissue interfaces, and typically performed 2–3 times to achieve a desired linewidth of ~20-30Hz for the whole brain. The 2D-CSI ^31^P volume of interest covered the whole brain with 8 slices of 3 cm thickness. Each slice had an 8x8 grid of voxels covering the entire slice. The slices were placed in the sagittal direction centered at midline. As a result, multiple 2D slices were acquired resulting in an 8x8x8 grid with a 24 cm FOV. The true acquired ^31^P-MRS voxel size was 3x3x3 cm for a volume of 27 cc/voxel. Zero-filling in k-space was done once in-plane resulting in a nominal voxel size of 1.5x1.5x3 cm for a volume of 6.75 cc/voxel.

Spectroscopic imaging parameters included 2048 points, 5000 Hz sweep width, 2000 ms TR, 2 averages, 55° flip angle at 51.3 MHz in the sagittal plane. After ^31^P-MRS was complete, a high-resolution, 8-slice sagittal T_2_-Fluid Attenuated Inversion Recovery sequence [FLAIR; 2200 ms TR, 12 ms TE, 780 ms inversion time (TI), 24 cm FOV, 0.94x0.94 mm] was acquired at exactly the same location with a 5 mm slice thickness at exactly the same location as each of the ^31^P-MRS CSI slices for reference.

#### Image analysis

MRS data was processed using XSOS (Dikoma Shungu/Xiangling Mao; Weill Cornell Medicine) written in IDL [45, 46] (Excelis Visual, Boulder, CO). Raw data was loaded into the program and processed using Hamming and Fermi k-space filters, a 7.5 mm center voxel shift, 20 Hz exponential filtering and zero-filling in time, x and y-domains prior to 3D Fast Fourier Transformation. A fixed first order phase of 4200° was applied to all spectra and data was automatically phased in zero order. Visualization of the PCr linewidth was done for the center voxel in each slice. The PCr peak was set at 0.0 ppm and the central spectrum set as a reference, and susceptibility corrections performed throughout the data set. Zero and first order phasing along with baseline correction was applied prior to peak area integration to all other voxels in the CSI data set by an experienced analyst (JPD). All spectra within a slice were analyzed contributing (8*8) 64 spectra in-plane * 4 slices for a total of 256 spectra per subject.

Peak area integration was performed around each of the seven well-resolved resonance peaks identified in **S1 Fig**: inorganic phosphate (Pi), phospho-creatine (PCr), total ATP (sum of α-ATP, β-ATP and γ-ATP moieties), phosphodiesters (PDE), and phosphomonoesters (PME). Peak areas of all reproducible resonances were found. The MRS processing software creates a 16x16 voxel image for each slice with the signal intensity equal to the peak area of the metabolite of interest in each voxel. The central 4 slices were co-registered in SPM to the 3D BRAVO sequence by using the 8-slice concordant image set acquired at the time of MRS. The integral of each metabolite resonance was calculated and expressed as a percent area of the total phosphorous signal in the corresponding spectrum. The ratios PCr/ATP, PCr/Pi, Pi/ATP, and PME/PDE were then computed, as this process allows for correction of many factors which can make absolute quantitation of ^31^P concentration difficult.

The 3D T_1_-Weighted BRAVO MRI scan was automatically processed using FreeSurfer 6.0 running under the Centos 7 Linux environment. Images were then processed in Statistical Parametric Mapping (SPM8) (http://www.fil.ion.ucl.ac.uk/spm/) implemented in Matlab 2021 (MathWorks; Natick,MA) [10, 28–32]. For each participant, we used the Normalized Mutual Information routine of SPM8 [47] to first align the T_1_ BRAVO sequence to the reference T_2_-FLAIR acquired at exactly the same location as the ^31^P-MRS CSI slices. The parametric metabolite MRS maps were then subsequently aligned with the skull stripped 3D T_1_-Weighted FreeSurfer scan. Volumetric MRI scans were resampled to a 256x256x256 matrix array whereas the parametric metabolite MRS maps were resized to 256x256 images but not interpolated beyond the original 16x16x8 matrix given partial volume errors would occur. The co-registered MRI and MRS maps were quantified using the subcortical gray and white matter segmentation tools implemented in FreeSurfer 6.0 and Desikan-Killiany Atlas-based regions of interest (ROI) [48, 49] applied to the aligned MRI for regional sampling.

We focused on brain regions with known metabolic vulnerability to metabolic aging and AD, including: frontal cortex (middle and superior frontal gyrus); PCC (posterior cingulate gyrus and precuneus); temporal cortex (inferior, middle and superior temporal gyrus); and medial temporal lobe (hippocampus, amygdala, entorhinal and parahippocampal gyrus) [26, 27]. The mean metabolite signal in each ROI was then computed using FreeSurfer. We also obtained total intracranial volume for normalization purposes.

#### Quality control

Both a qualitative and quantitative quality control (QC) evaluation of the results was performed. The qualitative evaluation included visual QC inspection of the original spectra and fitting results done by the same experienced analyst (JPD). If a metabolite peak was visually present, and its fit was assigned to the correct resonance—giving a minimal residue in the subtraction spectrum—the fitting result was accepted. For three participants, MRS spectra failed to fit properly due to excessively low signal-to-noise and had to be filtered out of the analysis. For all other participants, all peaks were visually identified as being present in adequate concentration in the center voxel of each slice prior to analysis. As a check, the quality of the PCr peak was always viewed and the peak shifted to 0.0 ppm as a reference. If the PCr peak was not clearly visible in the central voxel, the data in that slice was then not used in subsequent calculations. Only the last slice was excluded on less than 5% of all cases. However, the other three remaining slices were used for analysis.

Quantitative QC was performed using statistical analysis of metabolite ratios using data visualization and interquartile range methods. This identified three additional subjects with out-of-range values in at least one metabolite ratio in at least one region, typically medial temporal lobe, which also led to exclusion.

### Covariates

All analyses were adjusted by age (years) and total intracranial volume (cc). For exposures showing significant associations with outcome measures, we further examined midlife health indicators as confounders, including hypertension (systolic blood pressure ≥140 mm Hg or diastolic blood pressure ≥90 mm Hg and/or use of anti-hypertensive medications), hypercholesterolemia (plasma cholesterol ≥240 mg/dL), and hyperinsulinemia (HOMA-IR>1.8).

### Statistical analysis

Analyses were performed in SPSS v.25. Clinical measures were examined with general linear models or chi-squared tests as appropriate. Cohort characteristics are described using mean (standard deviation) and n, percentage (%), stratified by exposure group.

All brain imaging outcomes are continuous and were scaled to standard deviations and centered at 0. Standardized outcomes are reported in the results and figures. To enable comparison with previous studies and for clinical interpretability, unstandardized, multivariable adjusted metabolite measures are reported in the tables.

We used multivariable general linear models to test for differences in metabolite ratios by fixed effects of sex (men vs. women) and APOE4 status (APOE4+ vs APOE4-). For metabolites showing sex and APOE4 effects, we included a four-level exposure interaction term (levels: male APOE4+ vs male APOE4- vs. female APOE4+ vs female APOE4-). Regression models were constructed to obtain global P values to test for multivariable outcomes across brain regions for each metabolite, adjusting by the confounders listed above. A Bonferroni multiple comparisons adjustment was used to control the familywise error rate while simultaneously assessing the significance of pairwise comparisons between groups, at p<0.05.

Finally, we conducted a sensitivity analysis to examine the effect of APOE4 dose on regional outcomes showing combined effects of sex and APOE4 status. Univariate linear models were used to test for effects of sex, APOE-dose [non-carrier (APOE4-), heterozygote (APOE4+/-), homozygote (APOE4+/+)], and their interactions at p<0.05. Given the descriptive nature of this analysis and the smaller percentage of APOE4 homozygotes, Sidak tests were used to assess the significance of pairwise multiple comparisons between APOE4-dose groups within the male and female groups.

## Results

### Participants

We enrolled 233 participants for this study. Of these, 24 were excluded due to incidental findings on MRI (n = 5 small vessel disease or lacunar infarctions, n = 2 meningiomas, n = 1 mild hydrocephalus, n = 1 demyelination), MR artifacts (n = 2), incomplete ^31^P-MRS studies (n = 3) or out-of-range metabolite ratios (n = 3), and incomplete APOE4 data (n = 7). The remaining 209 participants were examined, including 166 women and 43 men with complete clinical exams, cognitive testing, APOE4 genotype, MRI and ^31^P-MRS exams.

Participant characteristics are shown in **Table 1**. Seventy-three women (44%) and 24 men (56%) were APOE4+. Groups were comparable for demographic measures. The male group included more cases of hypertension than the female group, whereas the female group included more cases of hypercholesterolemia than the male group (p<0.05), with no effects of APOE4 status (**Table 1**). Hypertension and high cholesterol were included as confounders in the analyses.

**Table 1:** Participant characteristics.

	Men		Women	
	APOE4-	APOE4+	APOE4-	APOE4+
N	19	24	93	73
Age, years	51(6)	51(8)	51(6)	51(6)
Education, years	18(2)	18(2)	17(2)	17(2)
Ethnicity, % white	74	75	72	86
AD family history, %	68	50	56	70
APOE4 dose, % homozygous	--	12	--	7
MoCA scores, unitless	29(1)	29(2)	29(2)	29(1)
Hypertension, %	21	17	7	8
Hypercholesterolemia, %	5	0	17	15
Hyperinsulinemia, %	11	13	7	13

Values are mean (SD) unless otherwise specified. Abbreviations: APOE4 carriers (APOE4+) vs non-carriers (APOE4-).

### Sex and APOE4 status effects on phosphorus metabolites

PCr/ATP measures by sex and APOE4 status are found in **Table 2**.

**Table 2:** Regional PCr/ATP by sex and APOE4 status.

Region	Group	APOE4 status	N	Mean	SE	95% C.I.	
Frontal	Men	APOE4-	19	3.162	0.118	2.929	3.395
		APOE4+	24	2.723*	0.103	2.519	2.926
	Women	APOE4-	93	2.530*	0.049	2.433	2.628
		APOE4+	73	2.469*	0.056	2.359	2.580
Temporal	Men	APOE4-	19	3.403	0.102	3.202	3.604
		APOE4+	24	3.182	0.089	3.007	3.358
	Women	APOE4-	93	3.134	0.043	3.050	3.218
		APOE4+	73	3.112*	0.048	3.016	3.207
PCC	Men	APOE4-	19	3.067	0.117	2.836	3.298
		APOE4+	24	2.921	0.102	2.719	3.123
	Women	APOE4-	93	2.797	0.049	2.700	2.893
		APOE4+	73	2.775	0.056	2.665	2.884
Medial temporal	Men	APOE4-	19	4.545	0.134	4.281	4.808
		APOE4+	24	4.246	0.117	4.016	4.475
	Women	APOE4-	93	4.173	0.056	4.063	4.283
		APOE4+	73	4.187	0.063	4.062	4.311

Unstandardized means, SE, adjusted by age and total intracranial volume, with 95% confidence intervals (CI). *Different from APOE4- men, p<0.05.

Abbreviations: APOE4 carriers (APOE4+) vs non-carriers (APOE4-); ATP, total adenosine triphosphate; PCC, posterior cingulate cortex and precuneus; PCr, phosphocreatine.

**Fig 1:**
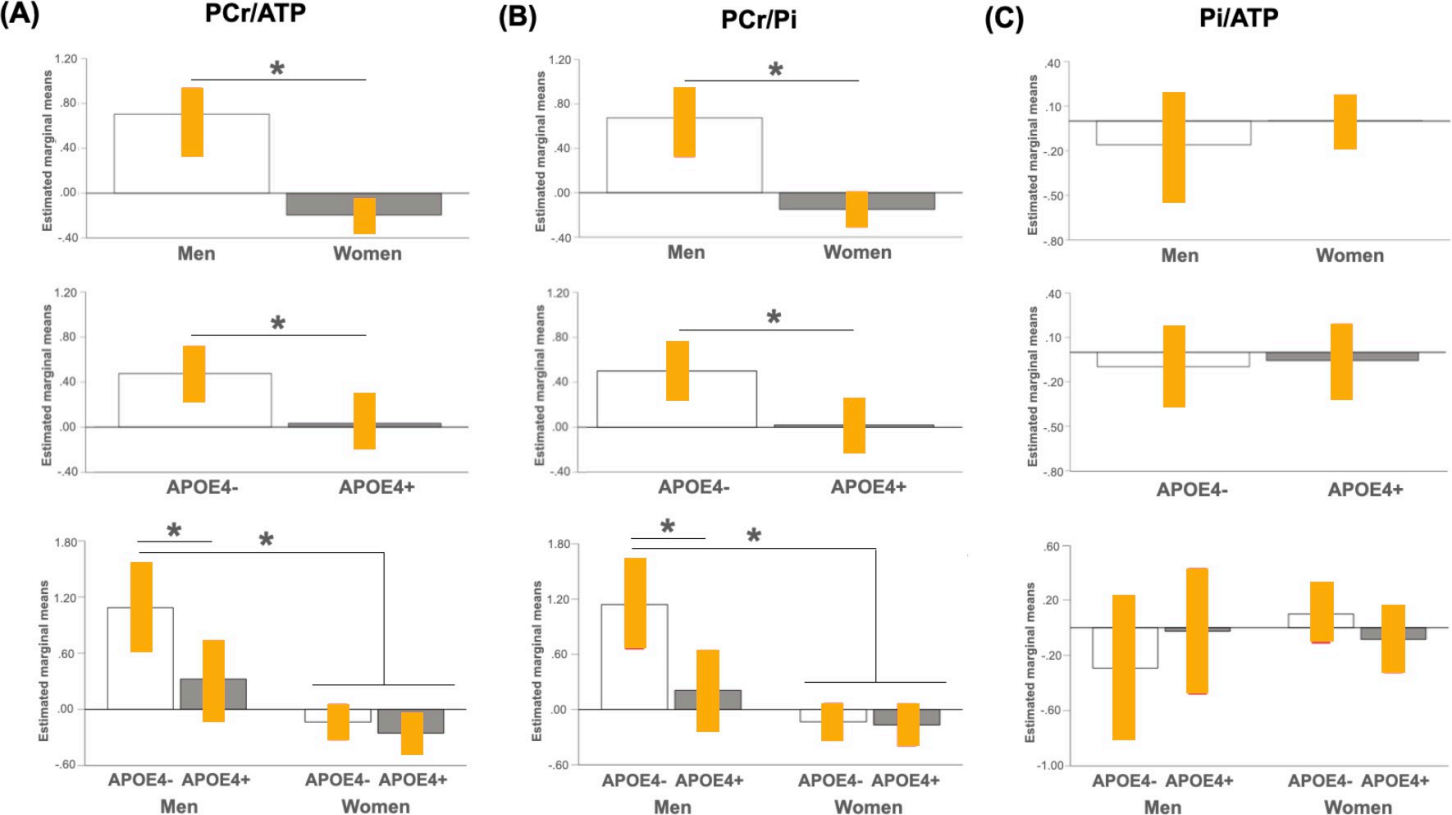
Effects of sex and APOE4 status on brain high-energy phosphates. Plots show mean high-energy phosphate (HEP) in frontal cortex for each pairwise comparison, with 95% confidence intervals (C.I.). *P<0.05, corrected for multiple comparisons. Abbreviations: APOE4-, non-carriers; APOE4+, carriers; ATP, total adenosine triphosphate; PCr, phosphocreatine; Pi, inorganic phosphate.

Adjusting by age and total intracranial volume, both sex (p<0.001) and APOE4 status (p = 0.040) were associated with PCr/ATP in AD-vulnerable regions. As shown in **Fig 1A,** on post-hoc analysis, women exhibited lower PCr/ATP than men in all regions examined, which remained significant after multivariable adjustment (p≤0.037), whereas effect of APOE4 was restricted to frontal cortex, with APOE4 carriers exhibiting lower frontal PCr/ATP than non-carriers (multivariable adjusted p = 0.007).

The interaction between sex and APOE4 status reached significance in frontal regions (multivariable adjusted p = 0.046), and was driven by both female groups and the APOE4+ male group exhibiting lower PCr/ATP than APOE4- men. No differences were observed between APOE4+ men and either female group (p≥0.234). As a result, a gradient was observed such as: APOE4- men > APOE4+ men = APOE4- women = APOE4+ women (**Table 2 and Fig 1A**).

Adjusting by age and total intracranial volume, both sex (p = 0.003) and APOE4 status (p = 0.023) were associated with PCr/Pi in AD-vulnerable regions **(Table 3 and Fig 1B)**. Following post-hoc analyses, women exhibited lower PCr/Pi than men in frontal, lateral and medial temporal regions (multivariable adjusted p≤0.010), though not in PCC. As with PCr/ATP, APOE4 effects were restricted to frontal cortex, with APOE4 carriers exhibiting lower PCr/Pi than non-carriers (multivariable adjusted p = 0.005; **Fig 1B**).

**Table 3:** Regional PCr/Pi by sex and APOE4 status.

Region	Group	APOE4 status	N	Mean	SE	95% C.I.	
Frontal	Men	APOE4-	19	16.708	1.043	14.652	18.765
		APOE4+	24	12.494*	0.911	10.698	14.290
	Women	APOE4-	93	11.107*	0.436	10.248	11.966
		APOE4+	73	10.978*	0.494	10.003	11.953
Temporal	Men	APOE4-	19	22.400	1.243	19.949	24.850
		APOE4+	24	19.423	1.085	17.284	21.563
	Women	APOE4-	93	18.492*	0.519	17.468	19.516
		APOE4+	73	18.254*	0.589	17.092	19.416
PCC	Men	APOE4-	19	13.169	1.050	11.099	15.239
		APOE4+	24	14.079	0.917	12.271	15.886
	Women	APOE4-	93	13.081	0.439	12.216	13.946
		APOE4+	73	12.388	0.498	11.407	13.369
Medial temporal	Men	APOE4-	19	31.251	2.419	26.481	36.021
		APOE4+	24	27.158	2.113	22.993	31.324
	Women	APOE4-	93	24.839	1.011	22.845	26.832
		APOE4+	73	23.213*	1.147	20.951	25.474

Unstandardized means, SE, adjusted by age and total intracranial volume, with 95% confidence intervals (CI).

*Different from APOE4- men, p<0.05

Abbreviations: APOE4 carriers (APOE4+) vs non-carriers (APOE4-); PCC, posterior cingulate cortex and precuneus; PCr, phosphocreatine; Pi, inorganic phosphate.

The interaction of sex and APOE4 status reached significance in frontal regions (multivariable adjusted p = 0.008), and was driven by both female groups and the APOE4+ male group exhibiting lower PCr/Pi than APOE4- men (p<0.001). There were no differences between APOE4+ men and either female group (p≥0.96) (**Table 3 and Fig 1B**). As a result, a gradient was noted such as: APOE4- men > APOE4+ men = APOE4- women = APOE4+ women.

**Fig 2:**
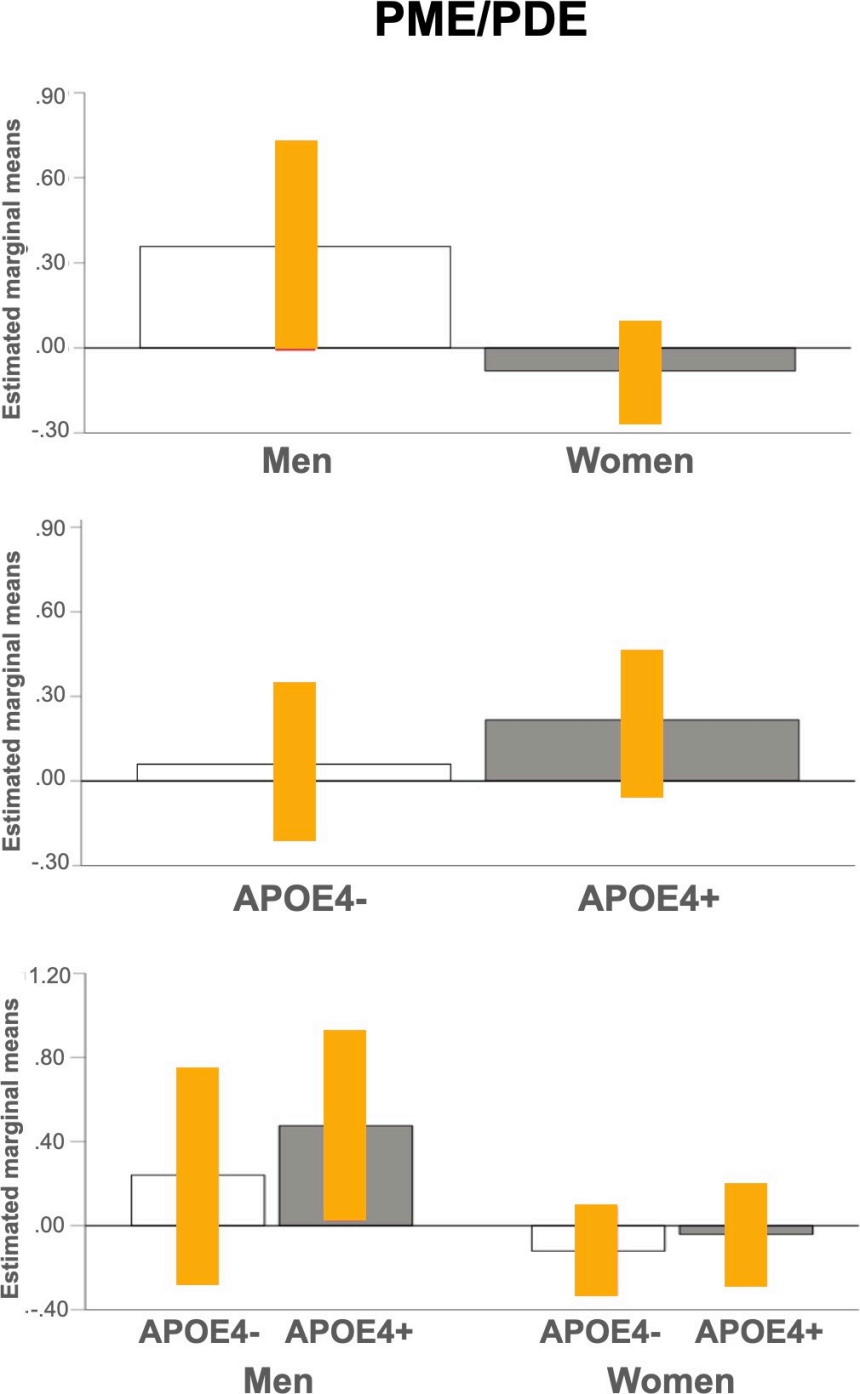
Effects of sex and APOE4 status on brain membrane phospholipids. Plots show mean PME/PDE measures in frontal cortex for each pairwise comparison, with 95% confidence intervals (C.I.). Abbreviations: APOE4-, non-carriers; APOE4+, carriers; PDE, phosphodiesters; PME, phosphomonoesters.

There were no significant effects of sex or APOE4 status on Pi/ATP (p>0.144; **Table 4 and Fig 1C)** or PME/PDE measures (p>0.211; **Table 5 and Fig 2**).

**Table 4:** Regional Pi/ATP by sex and APOE4 status.

Region	Sex	APOE4 status	N	Mean	SE	95% C.I.	
Frontal	Men	APOE4-	19	.434	0.036	0.363	0.505
		APOE4+	24	.464	0.032	0.402	0.526
	Women	APOE4-	93	.490	0.015	0.460	0.520
		APOE4+	73	.463	0.017	0.429	0.497
Temporal	Men	APOE4-	19	.487	0.022	0.444	0.530
		APOE4+	24	.514	0.019	0.476	0.552
	Women	APOE4-	93	.525	0.009	0.507	0.543
		APOE4+	73	.524	0.010	0.504	0.544
PCC	Men	APOE4-	19	.500	0.025	0.451	0.550
		APOE4+	24	.449	0.022	0.406	0.493
	Women	APOE4-	93	.463	0.011	0.442	0.484
		APOE4+	73	.472	0.012	0.448	0.495
Medial temporal	Men	APOE4-	19	.663	0.034	0.597	0.730
		APOE4+	24	.682	0.029	0.624	0.741
	Women	APOE4-	93	.727	0.014	0.699	0.754
		APOE4+	73	.734	0.016	0.703	0.766

Unstandardized means, SE, adjusted by age and total intracranial volume, with 95% confidence intervals (CI). Abbreviations: APOE4 carriers (APOE4+) vs non-carriers (APOE4-); ATP, total adenosine triphosphate; PCC, posterior cingulate cortex and precuneus; Pi, inorganic phosphate.

**Table 5:** Regional phosphomonoesters/phosphodiesters (PME/PDE) by sex and APOE4 status.

Region	Sex	APOE4 status	N	Mean	SE	95% C.I.	
Frontal	Men	APOE4-	19	1.754	0.118	1.521	1.988
		APOE4+	24	1.838	0.103	1.634	2.041
	Women	APOE4-	93	1.596	0.049	1.498	1.693
		APOE4+	73	1.634	0.056	1.524	1.745
Temporal	Men	APOE4-	19	2.956	0.118	2.724	3.188
		APOE4+	24	2.993	0.103	2.790	3.195
	Women	APOE4-	93	3.019	0.049	2.922	3.116
		APOE4+	73	2.855	0.056	2.745	2.965
PCC	Men	APOE4-	19	1.912	0.129	1.658	2.166
		APOE4+	24	1.627	0.113	1.404	1.849
	Women	APOE4-	93	1.629	0.054	1.522	1.735
		APOE4+	73	1.592	0.061	1.472	1.713
Medial temporal	Men	APOE4-	19	4.041	0.205	3.638	4.445
		APOE4+	24	4.270	0.179	3.918	4.623
	Women	APOE4-	93	4.196	0.086	4.028	4.365
		APOE4+	73	3.991	0.097	3.800	4.182

Unstandardized means, SE, adjusted by age and total intracranial volume, with 95% confidence intervals (CI). Abbreviations: APOE4 carriers (APOE4+) vs non-carriers (APOE4-); PME/PDE, phosphomonoesters/phosphodiesters.

### Sensitivity analysis

**Fig 3:**
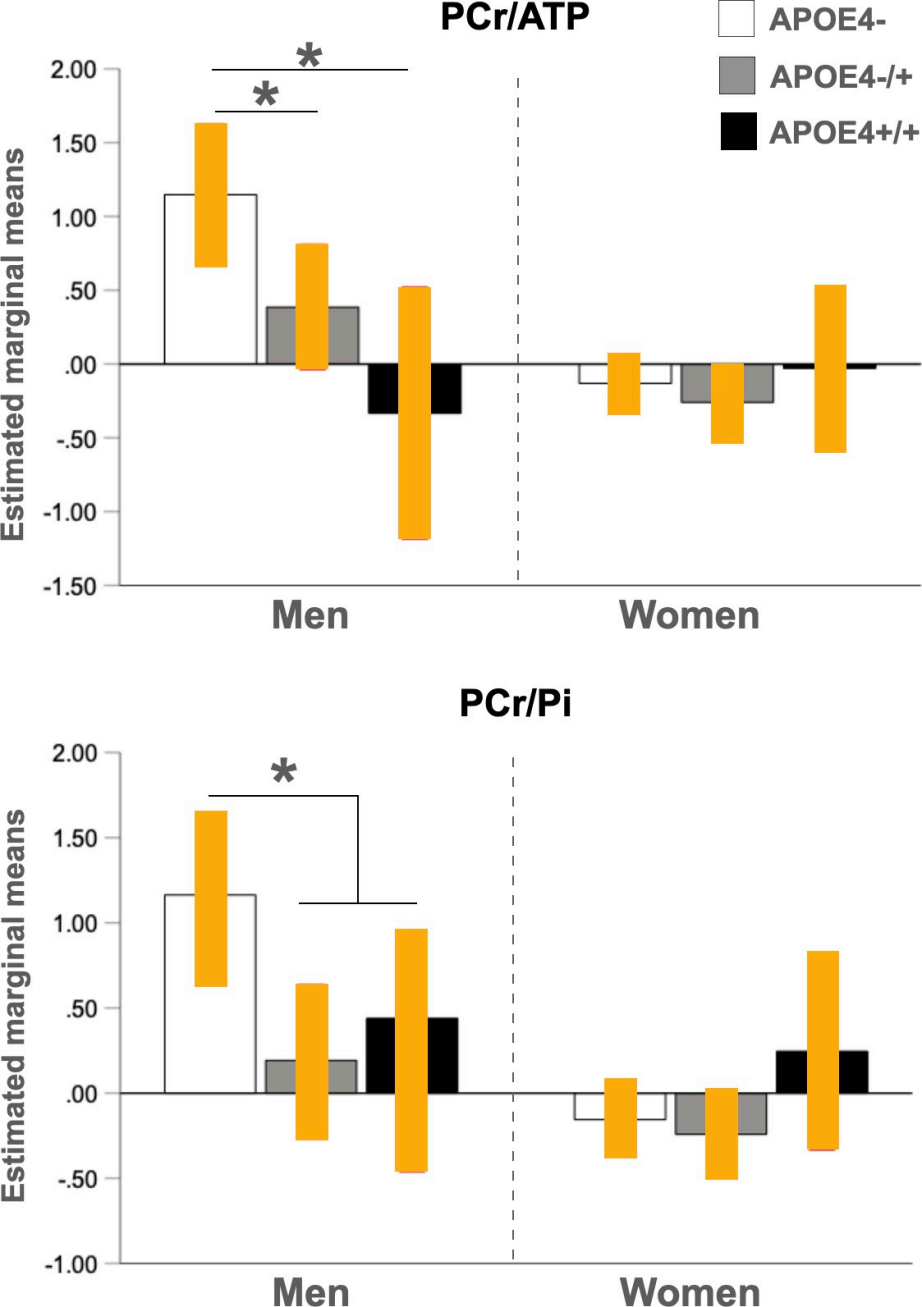
Effects of APOE4 dose on PCr/ATP and PCr/Pi. Plots show mean PCr/ATP and PCr/Pi measures in frontal cortex by APOE-dose group in men and women, with 95% confidence intervals (C.I.). *P<0.05, corrected for multiple comparisons. Abbreviations: APOE4-, non-carriers; APOE4-/+, heterozygous carriers; APOE4+/+, homozygous carriers; ATP, total adenosine triphosphate; PCr, phosphocreatine; Pi, inorganic phosphate.

Analysis of APOE4 dose was restricted to regional metabolites showing interaction effects of sex and APOE4 status, e.g. PCr/ATP and PCr/Pi in frontal regions. Albeit limited by the small number of APOE4 homozygotes, gender-related effects of APOE4 dose were observed for frontal PCr/ATP (p = 0.010). As shown in **Fig 3**, PCr/ATP levels were progressively lower by APOE4 dose among men, such as: APOE4-/- > APOE4+/- > APOE4+/+, whereas decreased PCr/ATP occurred in women regardless of APOE genotype.

## Discussion

This ^31^P-MRS study of cognitively normal midlife individuals at risk for AD demonstrates influences of sex and APOE4 status on mitochondrial PCr/ATP and PCr/Pi in AD-vulnerable regions, independent of age, total intracranial volume, and midlife health indicators. As compared to men, women exhibited lower PCr/ATP and PCr/Pi, reflecting higher brain ATP utilization and demand, respectively, in AD-vulnerable regions, independent of APOE4 status. Additionally, APOE4 carriers exhibited higher energy needs in frontal regions as compared to non-carrier status, which were driven by male APOE4 homozygotes.

AD is characterized by a long prodromal period of approximately 20 years, during which the disease progresses to clinically diagnosed dysfunction [17]. Identifying people at risk for AD while still in a modifiable transition state is likely critical for reversing or delaying disease progression. The three major AD risk factors—age, female sex, and APOE4 genotype—have a profound impact on brain bioenergetics [18], resulting in glucose hypometabolism [36], down-regulation of mitochondrial genes involved in OXPHOS [50], altered Aβ processing and decline in regenerative capacity [19–21]. In humans, both female sex and APOE4 genotype have been associated with reduced CMRglc in asymptomatic at-risk individuals [10, 28–36]. However, previous FDG-PET work was limited to assessment of the glycolytic pathway and did not provide direct information on brain mitochondrial activity.

Currently, no ^31^P-MRS studies have investigated APOE4 effects on brain phosphorus metabolites, and few studies have examined sex effects among cognitively normal individuals. Two studies tested for sex differences across the adult lifespan, reporting somewhat contrasting results. The largest study examined 125 individuals ages 20–85 years, reporting lower PCr/ATP (higher ATP utilization; averaged α-, β-, and γ-ATP resonances) and PCr/Pi (energy demand) in frontal, occipital, and temporal cortices in women as compared to men [51]. The other study, totaling 34 healthy volunteers ages 21–84 (20 men and 14 women), found no sex differences in α-, β-, or γ- nucleoside di- and tri-phosphate (NTP), Pi, or PCr measures [52]. The use of relative vs. absolute measures makes these studies difficult to compare. Nonetheless, from a methodological perspective, metabolite ratios are considered more reliable, as they are less prone to acquisition issues such as transmit and receive field variation, signal-to-noise ratio (SNR), and partial volume averaging concerns. By using metabolite ratios, a study of 74 cognitively normal, middle-aged post-menopausal women of known APOE4 status (48% APOE4 carriers) reported higher α-ATP utilization in temporal and frontal regions as compared to 45 age-controlled men [30], thus consistent with [51].

**Fig 4:**
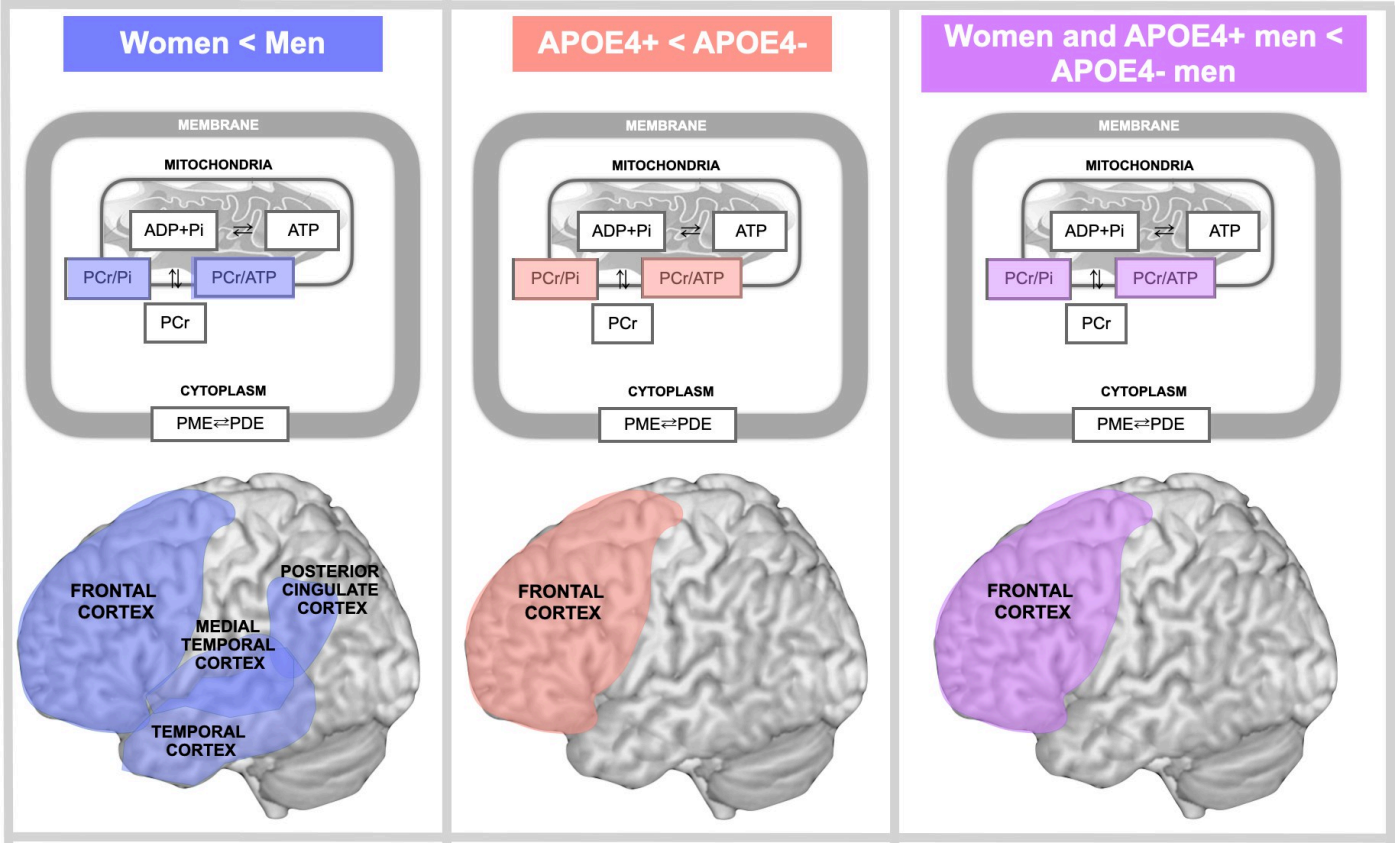
Schematic representation of sex and APOE4 status effects on midlife mitochondria phosphorus metabolites measured with ^31^P-MRS. All panels: Phosphorus metabolites assessed with ^31^P-MRS include phosphocreatine (PCr) rephosphorylated from creatine (Cr) using adenosine triphosphate (ATP) derived from chemical exchange of inorganic phosphate (Pi) and adenosine diphosphate (ADP) in mitochondria. Membrane phosphodiesters (PDE) and phosphomonoesters (PME) are also shown. Left panel: PCr/ATP and PCr/Pi are lower in women vs. men in frontal, temporal, medial temporal and posterior cingulate cortex (blue). Middle panel: PCr/ATP and PCr/Pi are lower in APOE4 carriers (APOE4+) vs. non-carriers (APOE4-) in frontal cortex (red). Right panel: PCr/ATP and PCr/Pi are lower in females and male APOE4 carriers vs. male non-carriers in frontal cortex (purple).

In the present study with a sample of over 200 middle-aged (40–65 year-old) individuals carrying established risk factors for AD, women exhibited lower PCr/ATP (higher ATP utilization; averaged α-, β-, and γ-ATP resonances) in AD-vulnerable regions including PCC, frontal, lateral and medial temporal cortices, and lower PCr/Pi (energy demand) in all regions except PCC as compared to age-controlled men. Additionally, we found male-specific associations between APOE4 carrier status and ATP utilization in frontal cortex, as men with two copies of APOE4 allele exhibited similar measures as the combined female group, but higher ATP utilization as compared to men with one APOE4 copy and to non-carriers. This effect is exemplified in **Fig 4**.

When interpreting present results relative to existing literature, it is important to note that prior ^31^P-MRS investigations of HEP metabolites in AD patients show generally mixed results. This is likely due to many studies being limited by small sample sizes, as well as to differences in brain regions examined, metabolite outcome reporting, and imaging methodology [53]. Older studies were also limited by acquisitions performed at 1.5 Tesla with only surface coil localization and lower SNR than 3.0 Tesla, resulting in limited coverage and introduction of inhomogeneous spin excitation [54]. Generally, older ^31^P-MRS reports on differences between AD patients and elderly controls provided mixed findings. Some reports indicated no differences in PCr/NTP or NTP (mostly comprised of the β-phosphate) in AD patients as compared to controls [55, 56], suggesting that NTP measures might be less sensitive to AD-related changes than ATP. While both lower [57] and higher [42, 58] γ-ATP have been reported in AD vs. controls, with no differences in α- or β-ATP observed in older studies [42, 57, 58], a recent study at 7 Tesla reported lower PCr/ATP, which averaged the α-, β-, and γ-ATP resonances, in AD patients as compared to healthy controls [40], whereas another study at 3 Tesla reported no differences [41]. Additionally, there is mixed evidence of lower [57] and higher PCr [41, 42] in AD patients compared to controls, or no differences [55, 56]. PCr levels may be reduced at the milder stages of AD but increase as dementia worsens [57].

AD-related alterations in PCr/Pi have also been noted, although results are again mixed with studies showing lower [40, 56] or higher [41] ratios in AD patients relative to controls. Additionally, results vary by brain region, as one report found lower PCr/Pi in frontal cortex of AD patients as compared to controls [56], while some studies have observed alterations in temporal cortex [40, 41] but not in frontal cortex [40].

Previous studies of age effects in cognitively normal individuals indicate that PCr, PCr/ATP and PCr/Pi generally increase with age [51, 52, 59]. However, these studies examined larger age ranges (20–85 years) as compared to 40–65 years in the current study. Moreover, in the only study that examined the combined effects of age and sex, women exhibited a nearly continuous increase of PCr/ATP with age *except* between the decades 20–29 and 30–39 years, 40–49 and 50–59 years, and 60–69 and 70–79 years [51]. In contrast, PCr/ATP in men only increased after age 70 [51]. On the other hand, PCr/Pi showed a slight decrease with age in women and an increase in men ages 70–79 years [51]. These results suggest presence of sex-specific turning points in brain ATP utilization, with earlier effects in women than in men, thus consistent with present findings. Overall, more studies with larger samples and follow-up evaluations are required to accurately characterize how HEP metabolite trajectories vary by sex and APOE status during normal aging and with AD.

Under normal aerobic conditions, mitochondrial ATP production increases to match increased energy demand, the former being reflected by a decrease in the PCr/ATP ratio [19]. Lower PCr/ATP and PCr/Pi ratios coupled with no difference in Pi/ATP ratio indicates that the sex and APOE4 differences are driven by reduced reserve in PCr to generate spare ATP. As such, as compared to men, women’s brains appear to require more effort to maintain stable energy production to meet increased energy demand in regions with known metabolic vulnerability to AD, further indicating that midlife hypometabolism might be an early female-specific indicator of prodromal AD [1, 18]. However, in frontal regions, male APOE4 homozygotes exhibited a similar metabolic range as women, identifying a male-specific APOE4-dose dependent earlier site of metabolic vulnerability. This is consistent with preclinical evidence for upregulation of glycolysis and TCA cycle pathways, and thus increased oxidative metabolism in humanized APOE4 mice [60].

Present findings support clinical and epidemiological studies indicating interactive effects of chromosomal sex and APOE4 allele on AD risk. Female APOE4 carriers are more likely than male carriers to develop AD, with a nearly 4- and 10-fold increased risk in women with one and two APOE4 alleles, respectively [1, 5, 14–16]. On the other hand, men heterozygous for APOE4 exhibit essentially no increase in AD risk, whereas those with two copies of APOE4 allele carry similar or greater risk for MCI and AD than homozygous women [5, 13, 15, 16]. Additionally, in women, carrying one APOE4 allele shifts the AD risk curve five years earlier, while two alleles shifts the curve to 10 years earlier in both women and men [61]. However, there is some evidence that sex-related differences in APOE4 effects on AD risk may change with disease progression. Overall risk for MCI is higher in men, whereas progression to AD occurs faster in women, at least partly in APOE4-dependent ways [13, 15, 16].

Biomarker studies provide further evidence for sex-related APOE4 effects on AD risk. Female APOE4 carriers with MCI or mild AD exhibit a higher burden of AD pathology, as well as greater CSF tau and tau/Aβ ratios than male APOE4 carriers with the same diagnosis [8, 9, 14, 62]. Among cognitively normal individuals, female APOE4 carriers exhibit greater hippocampal atrophy, cortical thinning, and brain hypometabolism [8–13] and higher Aβ deposition as compared to genotype-controlled men [10]. None of the above studies reported AD biomarker alterations in male APOE4 carriers. However, investigations of APOE4 dose effects in MCI patients show that APOE4 homozygous men exhibit smaller hippocampal volume than APOE4 homozygous women [11]. In another study of MCI and AD patients, while both female APOE4 heterozygotes and homozygotes showed increased tau deposition compared to female non-carriers, only male APOE4 homozygotes had increased tau deposition compared to male non-carriers [63].

Present results of bioenergetic alterations in frontal cortex of male APOE4 homozygotes is consistent with previous evidence that two copies of APOE4 are necessary to increase AD risk in men. Our data adds to previous literature by showing that metabolic abnormalities are evident already during the normal stages of cognition among men aged 40–65 years. Additionally, we identify the frontal cortex as an early site of APOE4-related metabolic compromise in males. Previous studies of asymptomatic APOE4 carriers identified the frontal cortex as a site of early metabolic vulnerability, especially in presence of a double APOE4 allele dose, for both men and women [33–36]. Greater hypometabolism in frontal cortex has been associated with conversion from MCI to AD [64], possibly due to Aβ deposition and tau acetylation with AD progression [65–67].

On the other hand, we didn’t find significant effects of APOE4 status on ATP requirements among women. This suggests that the effects of female sex may outweigh those of APOE4 status on at least some aspects of brain bioenergetics in midlife. Female sex is a known risk factor for reduced cerebral metabolic activity [1, 18], which has been linked to declines in 17β-estradiol during the menopause transition, or perimenopause [68]. In animal models, perimenopause decreases cerebral glucose utilization while triggering compensatory mechanisms to preserve ATP production by mitochondria, including increased breakdown of amino acids, fatty acids (β-oxidation), and ketone bodies [69–72]. However, continued reliance upon these pathways prompts white matter catabolism [70], Aβ dysmetabolism [24], and neurodegeneration [69]. As the majority of women in our studies were of perimenopausal or early postmenopausal age, higher brain ATP utilization in AD-vulnerable regions may reflect the compensatory shift to utilization of high energy lipid fuel observed in animals. As our study is underpowered to simultaneously examine effects of sex, APOE4 and menopause status, more work is warranted to characterize the independent and synergistic contributions of these AD risk factors on brain mitochondria function in midlife women.

Alternatively, as APOE4 effects are age-dependent [7, 73], and most clinical studies indicating a higher risk of AD among female APOE4 carriers were conducted in older populations, it is possible that APOE4 effects on ATP usage in women may become evident at older ages. For instance, a recent metabolomic analysis of older cognitively impaired and non-impaired individuals revealed alterations in serum metabolites associated with mitochondria energy production in post-menopausal women as compared to men, which were more pronounced among APOE4 carriers [74]. This suggests that APOE4 effects on mitochondria metabolism may become evident at older ages in women. It is also possible that metabolic changes in brain and periphery may follow a different time course.

We observed no significant effects of sex or APOE4 status on Pi/ATP and PME/PDE measures. To date, only one study examined sex differences in Pi/ATP (e.g. ATP hydrolysis), reporting lower ratios in temporal cortex and higher ratios in parietal cortex of women as compared to men across the adult lifespan [51]. Unlike our study, these results were not corrected for multiple comparisons. This suggests that sex-related PCr/ATP and PCr/Pi alterations may be of greater magnitude than, or precede Pi/ATP effects. Some studies also found Pi/ATP alterations, as well as changes in PME and PDE in AD patients as compared to healthy controls [43, 44], warranting further investigations of these biomarkers for the early detection of AD.

### Strengths and limitations

This study has several strengths. To our knowledge, this is the first ^31^P-MRS study to investigate the interactive effects of sex and APOE4 status on phosphorus metabolites in a large group of well characterized, cognitively normal middle-aged individuals carrying established risk factors for AD. All participants had clinical and cognitive exams, laboratory tests, and APOE4 assessments. We acquired multi-slice 2D-CSI ^31^P-MRS scans that cover the whole brain, thus mapping multiple voxels over a whole grid instead of large single voxels, which enabled us to simultaneously assess phosphorus metabolites in several AD-vulnerable regions. Results were significant after a stringent Bonferroni correction for multiple comparisons, and after multi-variable correction for age, midlife health indicators, and intracranial volume.

All participants in this study were cognitively intact, with cognitive performance within norms by age and education. As such, present results indicate that brain OXPHOS markers measured by ^31^P-MRS may precede changes in cognitive performance as related to AD risk. Mounting evidence indicates sex differences and APOE4 effects in the prevalence, symptomatology, and pathophysiology of AD [3, 4]. Despite experiencing an earlier onset and accelerated progression of AD pathology, women maintain clinically defined normal memory performance longer than men [75]. One theory suggests that sex differences in cerebral metabolism may explain, in part, the differences in disease progression, possibly delaying onset of clinical symptoms in women [76]. More work and longitudinal follow-ups are needed to characterize changes in brain ^31^P-MRS ATP utilization as a function of sex, APOE4, and cognitive status.

Our sample was comprised of middle-aged, highly educated, 75% white individuals, which limits the generalizability of our results. More studies are needed to replicate these findings in community-dwelling individuals randomly recruited from the population.

Finally, evaluation of metabolite peak areas using XSOS is operator-dependent and may introduce subjective errors due to phase distortions and baseline roll although the same 1^st^-order phase shift was applied to all subjects. While this might be a source of methodological bias, our technique is well validated [45, 46] and all fittings were performed by the same MRI physicist with over 20 years of experience in processing MRS data (JPD). As pointed out by others, ^31^P-MRS measurements are affected by many acquisition characteristics such as transmit and receive field variation, SNR, and partial volume averaging [51, 77]. In our study, all participants were scanned with the same dual tuned ^31^P/^1^H birdcage head coil with identical transmit and receive gains. Further, to overcome these issues and in keeping with the literature, we reported metabolite ratios, which are less sensitive to such factors.

We have not yet examined pH measures from our spectra. As some studies report pH alterations in AD patients as compared to controls [41, 58] or during normal aging [52, 59], this is of interest for future studies of AD risk, as well.

## Conclusions

Present ^31^P-MRS results in midlife individuals at risk for AD indicate complex interactions between chromosomal sex and APOE4 genotype on brain energy production and demand from mitochondria. Examination of metabolic heterogeneity can support identification of AD-specific pathways within at-risk subgroups, further advancing precision medicine for AD prevention.

## Supporting information

S1 Fig ^31^P-MRS spectra of two representative participants. Seven well-resolved resonance peaks were identified, including the HEP molecules phosphocreatine (PCr), adenosine triphosphate (α-ATP, β-ATP and γ-ATP), and inorganic phosphate (Pi); and membrane phosphomonoesters (PME) and phosphodiesters (PDE). P_i_ and phospholipids are located to the left of PCr. Resonant peaks from the three phosphate groups of ATP (γ-, α-, and β-ATP from left to right) are located to the right of PCr. A spectrum from the frontal lobe is shown in (A) a 55 year-old woman and (B) a 55 year-old man.
(TIF)
