# Supplementary figures and images for: Effects of sex and APOE ε4 genotype on brain mitochondrial high-energy phosphates in midlife individuals at risk for Alzheimer’s disease: A 31Phosphorus MR spectroscopy study
Source: PLoS One. 2023 Feb 14;18(2):e0281302. doi: 10.1371/journal.pone.0281302 (PMC9928085; doi:10.1371/journal.pone.0281302)

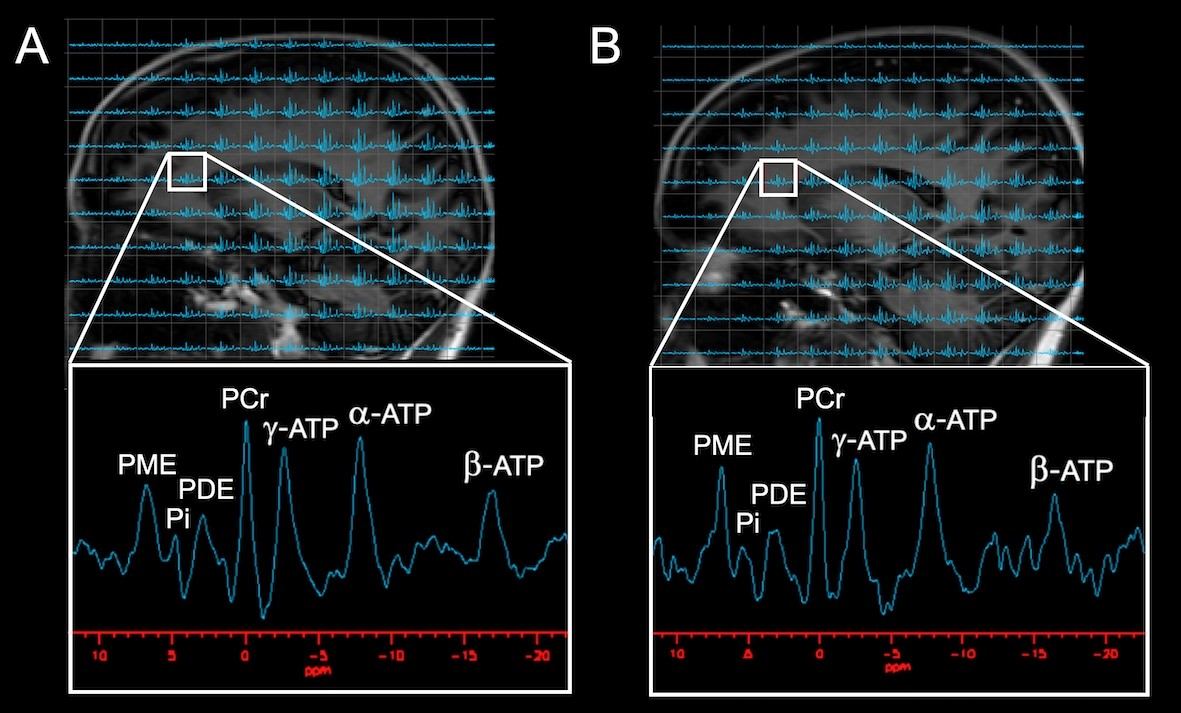

Supplement: S1 Fig — Seven well-resolved resonance peaks were identified, including the HEP molecules phosphocreatine (PCr), adenosine triphosphate (α-ATP, β-ATP and γ-ATP), and inorganic phosphate (Pi); and membrane phosphomonoesters (PME) and phosphodiesters (PDE). Pi and phospholipids are located to the left of PCr. Resonant peaks from the three phosphate groups of ATP (γ-, α-, and β-ATP from left to right) are located to the right of PCr. A spectrum from the frontal lobe is shown in (A) a 55 year-old woman and (B) a 55 year-old man. (TIF) [file pone.0281302.s001.tif]
